# Hemodynamic and neuronal contributions to low-frequency vascular oscillations in a preclinical model of Alzheimer’s disease

**DOI:** 10.1117/1.NPh.12.S1.S14615

**Published:** 2025-07-22

**Authors:** Shannon M. O’Connor, Runchong Wang, Paul S. Sharp, Osman Shabir, Kira Shaw, Michael Okun, Clare Howarth, Chris Martin, Jason Berwick

**Affiliations:** a University of Sheffield, School of Psychology, Faculty of Science, Sheffield, United Kingdom; b Medicines Discovery Catapult, Macclesfield, United Kingdom; c University of Sheffield, School of Medicine and Population Health, Division of Clinical Medicine, Sheffield, United Kingdom; d University of Sheffield, Neuroscience Institute, Sheffield, United Kingdom; e University of Nottingham, School of Life Sciences, Nottingham, United Kingdom; f University of Sheffield, Healthy Lifespan Institute, Sheffield, United Kingdom

**Keywords:** vasomotion, Alzheimer’s disease, neurovascular coupling

## Abstract

**Significance:**

Vasomotion, a temporal oscillation in vascular diameter centered around 0.1 Hz, may be altered in Alzheimer’s disease (AD), with both increases and decreases reported.

**Aim:**

We aimed to better characterize vasomotion *in vivo*, assess its feasibility as an early biomarker for vascular dysfunction in AD, and determine the relationship of vasomotion to underlying neuronal activity.

**Approach:**

Low-frequency (0.06 to 0.2 Hz) oscillations (LFOs) in the cerebral arteries of anesthetized 9- to 12-month-old J20-AD (n=12) and wild-type (n=10) mice were extrapolated from hemodynamic data obtained using 2D optical imaging spectroscopy (2D-OIS). Changes in LFO power were determined after an inspired gas challenge and compared between groups. Simultaneously gathered multi-unit neuronal activity data were used to determine whether LFOs were independent of neural activity.

**Results:**

LFOs increased as inspired oxygen was reduced, but the change in LFO power did not differ between groups. LFOs were found to be driven by neuronal activity, suggesting that they represent spontaneous low-frequency neurovascular coupling rather than vascular-only derived activity.

**Conclusions:**

Arterial LFOs obtained by 2D-OIS were not a suitable metric to distinguish anesthetized J20-AD males from healthy male controls. Furthermore, hemodynamic oscillations occurring within the same frequency range as vasomotion may reflect underlying neuronal activity.

## 1 Introduction

Vascular dysfunction is a commonly observed occurrence in both Alzheimer’s disease (AD)^1^ and aging.^2^ In some cases, alterations in vascular function have been observed decades before the onset of AD, suggesting that the earliest marker of AD progression may be vascular dysregulation.^3^ The neurovascular degeneration hypothesis of AD postulates that neurovascular dysfunction can lead to the onset or worsening of AD dementia through factors such as blood-brain barrier dysfunction and impaired cerebral blood flow (CBF), which can result in the development of tau-related pathology and a build-up of amyloid-beta peptide (Aβ),^4^^,^^5^ considered to be neuropathological hallmarks of AD.^6^

To ensure proper functioning of neurons in the brain, an adequate supply of blood is required, which is delivered via a vast vascular network of veins, arteries, venules, arterioles, and capillaries. Neurovascular coupling (NVC), or functional hyperemia, refers to the relationship between neuronal activity and CBF, whereby increases in neuronal activity drive an increase in CBF to the active area. This rapid redirection of blood flow to areas of neuronal activation ensures a targeted and uninterrupted flow of blood that is essential for brain viability.^7^ Despite a need for a continuous supply of glucose and oxygen, the brain lacks fuel stores^8^ and therefore relies on the concerted action of the cells within the neurovascular unit to maintain brain homeostasis and ensure that adequate energy and nutrients are available when required. It has been reported that deficits in NVC are present in AD, in both human patients and preclinical models of the disease,^9^^–^^13^ but there remains some uncertainty over whether NVC deficits are present in mouse models used in the study of AD.^14^^–^^16^

Another aspect of vascular function that may be altered in disease and aging is vasomotion. This is a rhythmic oscillation in vascular diameter, which peaks around 0.1 Hz.^17^^–^^19^ The physiological importance of vasomotion remains somewhat unclear; however, higher amplitudes and prevalence of vasomotion have been reported in conditions associated with compromised oxygen delivery,^20^^–^^23^ suggesting that the role of vasomotion may be of a protective nature.^24^ It has been postulated that the oscillatory blood flow produced by vasomotion may achieve better tissue oxygenation than that obtained from a steady flow of blood.^25^ Furthermore, it has been reported that hypoxic conditions have resulted in an increase in arteriolar vasomotion in an un-anesthetized hamster skinfold preparation, whereas inducing hyperoxia (using 100% $O_{2}$) resulted in a reduced frequency of vasomotion.^26^ Studies investigating vasomotion in the context of AD have been relatively limited; however, both enhancements^27^^,^^28^ and reductions^29^ in vasomotion have been reported in human patients, as well as impairments observed in mice expressing APOE4, a genetic risk factor for AD.^30^ Vasomotion has also been theorized to contribute to the driving force of solute clearance from the brain,^31^^,^^32^ and thus, impairments in this process might contribute to or aggravate progression of pathology in AD by hindering the removal of Aβ. Thus, a better understanding and characterization of vasomotion in AD is of significant importance. Classically defined vasomotion (i.e., of a purely vascular origin) can occur in any tissue of the body,^20^^,^^23^^,^^26^^,^^27^ and Hudetz et al., among others, showed the same oscillation in the brain.^18^^,^^33^ The term “vasomotion” has been used to describe two separate phenomena, a 0.1 Hz oscillation originating from intrinsic vascular mechanisms, or a mixture of neural-induced and neural-independent mechanisms. For the current study, we refer to vasomotion resulting from vascular-only derived activity as “neural-independent” vasomotion.

To enable the investigation of vasomotion and its physiological basis in health and disease, various *in vivo* mesoscale imaging studies have converted blood volume changes into the frequency domain and categorized blood volume change occurring around 0.1 Hz as vasomotion (for a review on this issue, see Das et al.^34^). However, this categorization may not be accurate when investigating cerebrovascular vasomotion, as neural activity also occurs at 0.1 Hz. Consequently, the low-frequency vascular oscillation may represent a blend of vasomotion and spontaneous NVC (i.e., neurally induced blood volume changes that are distinct from vasomotion). Furthermore, the role of neural activity in low-frequency oscillation remains largely unknown. Mateo et al.^35^ found that the pial vessel diameter covaries with slow changes in the amplitude of high-frequency LFP (gamma band: 30 to 80 Hz) in the vibrissa area, and activation of layer 5b pyramidal neurons at the same frequency leads to pial vessel diameter change at 0.1 Hz. Winder et al.^36^ also found blood volume change correlated with gamma-band LFPs in the somatosensory cortex. However, when local neural activity was substantially suppressed by muscimol, no significant changes in low-frequency vessel diameter were observed compared with the control group.

Existing studies thus provide evidence for both neural-induced and neural-independent low-frequency blood volume oscillations in wild-type (WT) mice, whereas the neural basis of low-frequency blood volume oscillations in mouse models of AD remains largely unexplored. The current study uses data collected from the transgenic J20-AD mouse model, a widely used model in preclinical studies of Alzheimer’s disease. The J20-AD mouse model overexpresses human amyloid precursor protein (APP) with familial AD mutations^37^ and recapitulates AD-like phenotypes such as synaptic loss,^38^ impairments in cognition,^39^ and plaque pathology from 6 months of age onward.^40^

As vasomotion could potentially be a biomarker of the vascular dysfunction that may occur in AD, the current study aimed to investigate low-frequency hemodynamic oscillations (LFOs) occurring in anesthetized J20-AD mice compared with WT C57Bl/6 controls and to determine whether these oscillations are independent of neural activity. We tested the hypothesis that a respiratory challenge in the form of an inspired gas transition from 100% oxygen to medical air (21% $O_{2}$) will be sufficient to drive increases in vasomotion oscillations.

## 2 Materials and Methods

To assess whether LFOs in cerebral blood volume differ between diseased and healthy states, data from experiments in which the hemodynamic responses and neurovascular function of mice were recorded using 2D optical imaging spectroscopy (2D-OIS), and electrophysiology were analyzed. 2D-OIS offers measurements of changes in total hemoglobin (HbT), akin to changes in cerebral blood volume and oxygenation, with high temporal and spatial resolution.^41^ When used alongside electrophysiology, this approach offers a concurrent measurement of hemodynamic and neural activity. Previously, we developed a chronic mouse preparation in which a thinned cranial window implant enabled repeated hemodynamic imaging followed by a single acute experiment in which 2D-OIS and electrophysiological silicon-probe recording were performed simultaneously. Data from these experiments have been published previously (see Shabir et al.^15^ and Sharp et al.^16^); however, no analysis was performed to explore LFOs or vasomotion in data collected from these animals.

### 2.1 Animals and Experimental Paradigm

Changes in the blood volume and oxygenation of anesthetized 9- to 12-month-old J20-AD (MMRRC Stock No: 34836-JAX) and WT C57Bl/6 controls [N=23 mice, (n=13 J20-AD, n=10 WT), males] were analyzed. Plaque pathology and impairments in long-term memory have been confirmed to be present by 9 months of age in this mouse model using an age-matched sibling cohort^40^ to the animals in the current study. All animals were anesthetized with fentanyl-fluanisone (Hypnorm, Vetapharm Ltd., New South Wales, Australia), midazolam (Hypnovel, Roche Ltd., Basel, Switzerland), and sterile water (ratio of 1:1:2 by volume; 7  mL/kg, i.p.) for surgery and imaging, in addition to 0.3% to 0.8% isoflurane in 100% oxygen for maintenance, as described previously by Sharp et al.^16^ Our established anesthetic regime has been determined to enable evoked hemodynamic responses resembling those in the awake state.^42^

All animals underwent three chronic imaging sessions, beginning ∼1 week after thinned cranial window surgery. Each session was separated by ∼30 days. The animals then underwent a final acute (fourth) imaging session, in which a 16-channel electrode (100  μm spacing, site area ${177\;\mu m}^{2}$, 1.5 to 2.7 MΩ impedance; Neuronexus Technologies, Ann Arbor, Michigan, United States) was inserted into the right whisker barrel cortex (WBC), which allowed for simultaneous measurement of neural activity and hemodynamic function. Each 2D-OIS session consisted of eight experiments performed in the same order.

**Exp1**: Sensory stimulation while breathing 100% oxygen. The experiment consisted of 30 trials of 25 s each, with whisker stimulation administered 5 s after the start of each trial. Whiskers were mechanically deflected using a plastic T-shaped stimulator for 2 s to evoke a hemodynamic response in the somatosensory cortex of the mouse. 2D-OIS lasted 750 s.
**Exp2**: Repeat of experiment 1 to ensure preparation and recording stability.
**Exp3**: Mild gas challenge: transition of breathing gas from 100% oxygen to normal air. 2D-OIS lasted 750 s with the transition to air occurring after 105 s.
**Exp4**: Sensory stimulation while breathing air. Paradigm is the same as experiment 1, except the animal was breathing air rather than oxygen.
**Exp5**: Long-duration sensory stimulation while breathing air. The experiment consisted of 15 trials of 70 s each, with whisker stimulation administered 10s after the start of each trial. 2D-OIS lasted 1050 s.
**Exp6**: Mild gas challenge: transition of breathing gas from air to 100% oxygen. 2D-OIS lasted 750 s with the transition to air occurring after 105 s.
**Exp7**: Long-duration sensory stimulation while breathing oxygen. Paradigm is the same as experiment 5, except the animal was breathing oxygen rather than air.
**Exp8**: Major gas challenge in the form of hypercapnia. The animal was switched from breathing 100% oxygen to 90% oxygen and 10% carbon dioxide. The duration of the challenge was 250 s.

The current study analyzed data from experiments 3 and 6, in which mice underwent a mild gas challenge in which inspired gas was switched from 100% $O_{2}$ to medical air (21% $O_{2}$) during the course of the experiment (see Sharp et al.,^16^ for more detailed experimental outline). In acute sessions, in which electrophysiology occurred alongside 2D-OIS, imaging experiments started 30 min after the electrode had been inserted. All animal procedures were performed with approval from the UK Home Office in accordance with the guidelines and regulations of the Animal (Scientific Procedures) Act 1986 and were approved by the University of Sheffield ethical review and licensing committee.

### 2.2 Measurement of Arterial LFOs

**Fig. 1:**
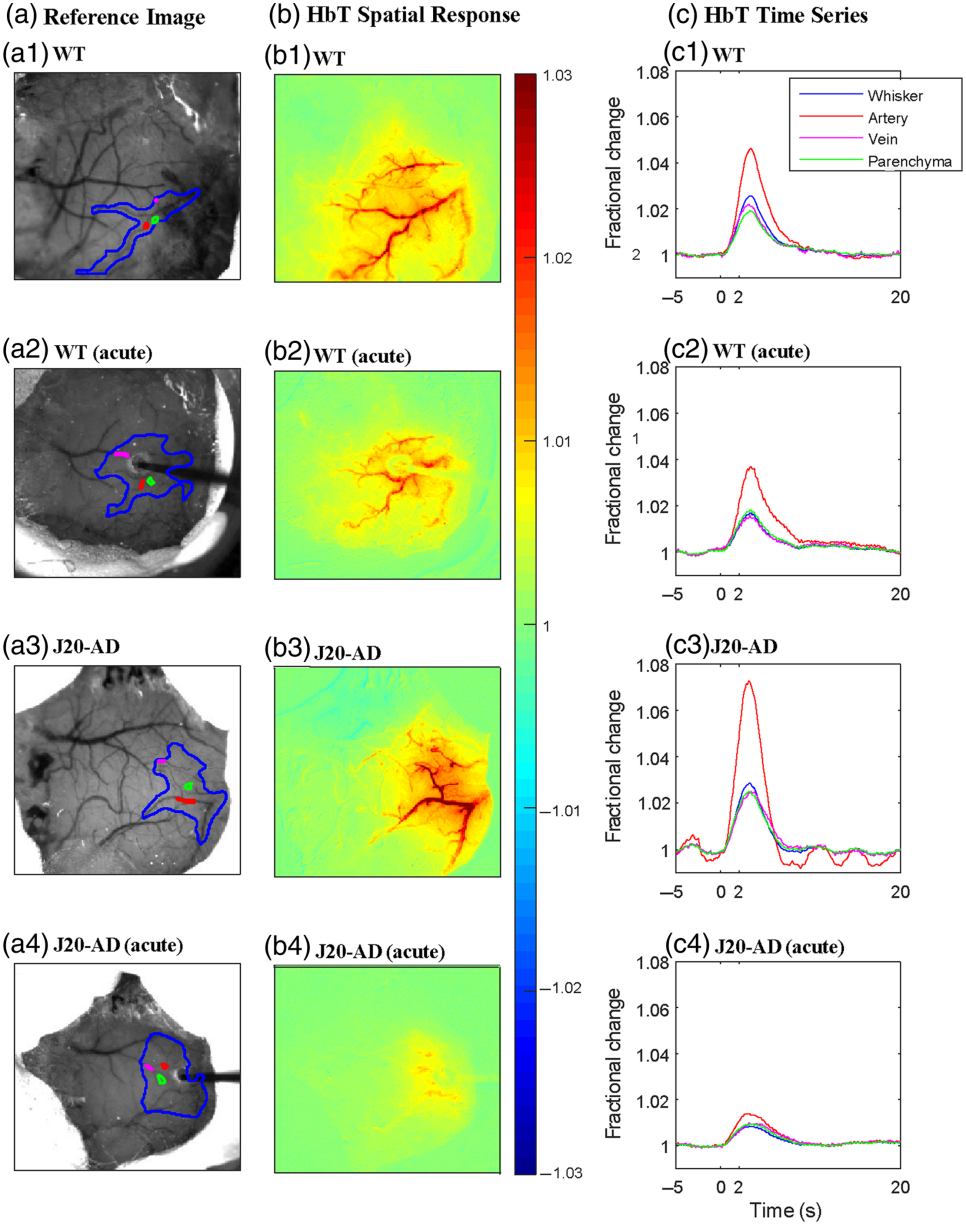
Region of interest selection and HbT response to whisker stimulation. A reference image of the thinned cranial window over the somatosensory cortex with four regions of interest (ROIs) selected: whisker region (blue), artery (red), vein (pink), and parenchyma (green) for (a1) WT, (a2) WT in the acute session (i.e., with electrode present), (a3) J20-AD, and (a4) J20-AD in the acute session (i.e., with electrode present). The corresponding HbT spatial response to a 2 s mechanical whisker stimulation of (b1) WT, (b2) WT in the acute session, (b3) J20-AD, and (b4) J20-AD in the acute session. HbT time series for (c1) WT, (c2) WT in the acute session, (c3) J20-AD, and (c4) J20-AD in the acute session. Whisker stimulation occurred at 0 s (after a 5 s baseline) for a 2 s duration. The color bar represents fractional change.

Recordings of changes in HbT were used to obtain a measure of LFOs occurring in four regions of interest (whisker barrel cortex [WBC], artery, vein, and parenchyma, Fig. 1). WBC location was determined based on the HbT activation map from a stimulation experiment in which whiskers were mechanically deflected using a plastic T-shaped stimulator to evoke a hemodynamic response in the somatosensory cortex of the mouse. Data were imported to MATLAB (R2022a; MathWorks, Natick, Massachusetts, United States^43^) for analysis. HbT data from 375 to 750 s of experiments 3 and 6 were used to assess LFOs in each inspired gas condition. A high-pass filter with a cutoff frequency of 0.01 Hz was applied to remove low-frequency noise. A fast Fourier transform (FFT) was conducted to obtain the data in the frequency domain for analysis. Oscillations within 0.06 to 0.2 Hz, a range typically associated with vasomotion,^44^^,^^45^ were summed for each animal in both the oxygen-breathing condition (Exp 6) and the air-breathing condition (Exp 3). As the strongest response was expected to occur in the artery, the main focus of the current study was on this region.

### 2.3 Downsampling Multi-Unit Activity (MUA)

Neural activity was recorded using a 16-channel electrode implanted in the whisker barrel cortex, concurrently with 2D-OIS HbT measurements. Signals from channels 3 to 8 were high-pass filtered, and events exceeding 1.5 standard deviations from the mean were identified and counted as spikes. Then, MUA was downsampled to match the 8 Hz sampling frequency of the HbT recordings. Downsampling was achieved by identifying the MUA timestamps that were closest to the HbT timestamps and calculating the total MUA activity within each 0.125 s interval. This approach preserved temporal alignment between MUA and HbT data.

#### 2.3.1 Kernel analysis of MUA and HbT

The linear relationship between MUA and HbT, representing NVC, was modelled using a kernel-based approach.^46^ The relationship is expressed as

H(t)=K*M(t), (1)

where H(t) is the predicted HbT, M(t) is the MUA, ∗ denotes the convolution operator, and K is the kernel.

**Fig. 2:**
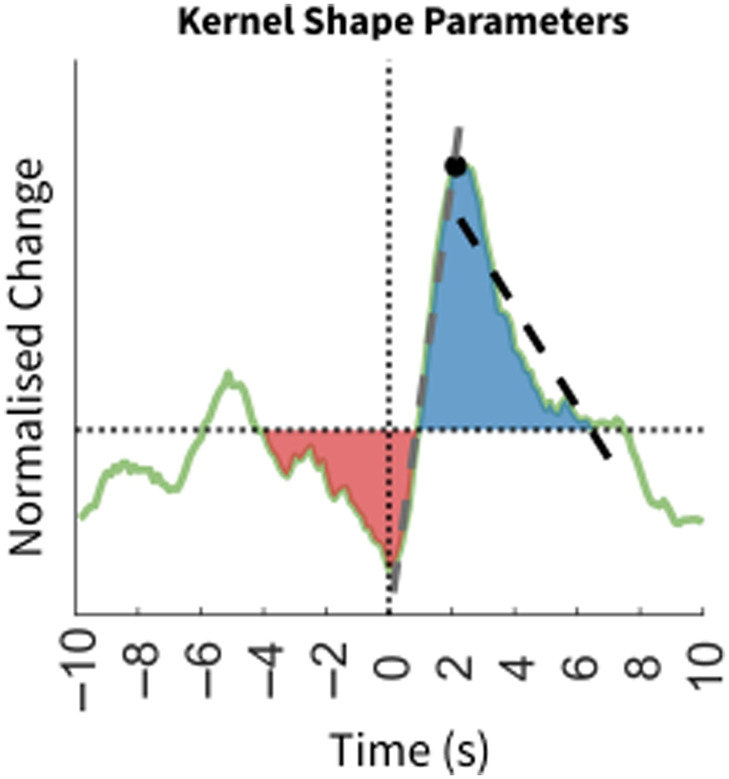
Kernel shape parameters. An example of a kernel. The red and blue region highlights the region before and after, respectively. The grey dashed line represents the slope to peak (from time 0 s to the peak), whereas the black dashed line represents the slope to baseline (from the peak to the x-axis). The black dot represents the peak.

The correlation between the measured HbT and the predicted HbT was computed to evaluate how effectively MUA explains HbT fluctuations. The kernel was normalized, and five key parameters were defined to describe the shape of the kernel (see Fig. 2):

##### Peak

The maximum value of the kernel occurring after time 0 s, representing the highest HbT response following neural activity.

##### Slope to peak

The best-fit line between time 0 s and the peak, reflecting how rapidly HbT changes in response to neural activity.

##### Slope to baseline

The best-fit line between the peak and when the kernel intersects with the baseline, representing how quickly HbT returns to baseline after neural activity.

##### Region before

The area under the kernel curve before the intersection between kernel and baseline, indicating the amount of HbT decrease preceding neural activity.

##### Region after

The area under the curve after time 0 s, capturing the total HbT increase associated with neural activity.

#### 2.3.2 Peak-triggered analysis

To confirm the kernel analysis result, we conduct an additional analysis. For both WT and AD groups in air and oxygen, we manually selected HbT peaks and then extracted the hemodynamic time series [i.e., HbT, oxyhemoglobin (HbO), and deoxyhemoglobin (HbR)] 10 s prior and 10 s post this peak [WT oxy (n=10, average number of peaks=38±2.9  sem), WT air (n=10, average number of peaks 37±2.1  sem), AD oxy (n=12, average number of peaks=47±3.5  sem), and AD air (n=12, average number of peaks 51±4.5  sem)]. All trials were averaged to create a mean hemodynamic time series across animals. To understand if there was a neural component to vasomotion, we took all the time points used to create these hemodynamic vasomotion trials to extract time-matched neuronal responses from the MUA data.

### 2.4 Statistical Analysis

#### 2.4.1 Analysis of HbT data

Analysis was conducted to determine the effect of group (J20-AD or WT C57Bl/6 control) and inspired gas (oxygen or air) on the power of LFOs in HbT in the artery ROI. Analysis of the chronic sessions included N=21 mice (n=11 J20-AD and n=10 C57Bl/6 controls), and each mouse contributed between 1 and 4 sessions to the analysis. To account for multiple measurements taken from the same subject, data were analyzed using a linear mixed model (LMM) (R version 2024.04.2+764, lme4 package, Bates et al.^47^) to account for random (animal ID, session ID) and fixed (group) effects on LFO power in the oxygen and air breathing conditions. Where post-hoc tests were performed, Tukey’s method for multiple comparisons was applied.

Previous literature has shown that the electrophysiological preparation can affect the cerebral hemodynamic responses due to a cortical spreading depression (CSD) as a result of electrode insertion into the cortex, resulting in a sustained reduction in HbT.^15^ Therefore, acute sessions in which an electrode was in place were analyzed separately. Two additional mice underwent acute imaging, resulting in N=23 (n=13 J20-AD and n=10 C57Bl/6 control) mice, with each animal contributing one session for each inspired gas condition. However, one acute session was removed from the analysis due to noise, which was impacting the quality of HbT and MUA measurements. All other sessions from all animals were included in the analysis. Therefore, N=22 (n=12 J20-AD and n=10 C57Bl/6 control) mice were included in the analysis. A mixed analysis of variance (ANOVA) was conducted to determine the effect of group (J20-AD or WT C57Bl/6 control) and inspired gas (oxygen or air) on the power of LFOs in HbT in the artery ROI. An a-level of 0.05 was considered to be statistically significant for both analyses.

#### 2.4.2 Analysis of HbT and neural data

Analysis was then conducted to explore the relationship between MUA and HbT. An electrode implant was present only in the final terminal experiment for each animal; thus, each animal only contributed one imaging session to the analysis. A mixed model ANOVA was conducted on LFP band power, parameters of kernel shape and kernel prediction across groups, and inspired gas conditions (R 2024.04.2+764, rstatix package; Kassambara^48^). The assumptions of normality and homogeneity of variance were assessed using Q–Q plots and Levene’s test, as implemented in the rstatix package (Kassambara^48^). The assumption of homogeneity was violated for the increasing slope. Therefore, a robust ANOVA (WSR2 package; Mair and Wilcox^49^) was used for increasing slope. An a-level of 0.05 was considered statistically significant. A simple effects test was conducted for any variables with a significant interaction, and a Bonferroni correction was applied to correct for multiple comparisons for the five kernel parameters and five LFP power bands.

For both analyses, preliminary screening of the data indicated a skewed distribution of LFO power, and as such, a log transformation was performed to improve normality of the distribution to increase the robustness of the applied statistical model and meet required assumptions. Researchers were blinded to animal group information (J20-AD or WT control) while statistical tests were performed. Where possible, the same arterial segment of each animal was used for both chronic and acute analyses.

## 3 Results

### 3.1 Arterial Low-Frequency Oscillations Are Unchanged in J20-AD Mice Compared with WT Controls (Chronic Imaging Sessions)

**Fig. 3:**
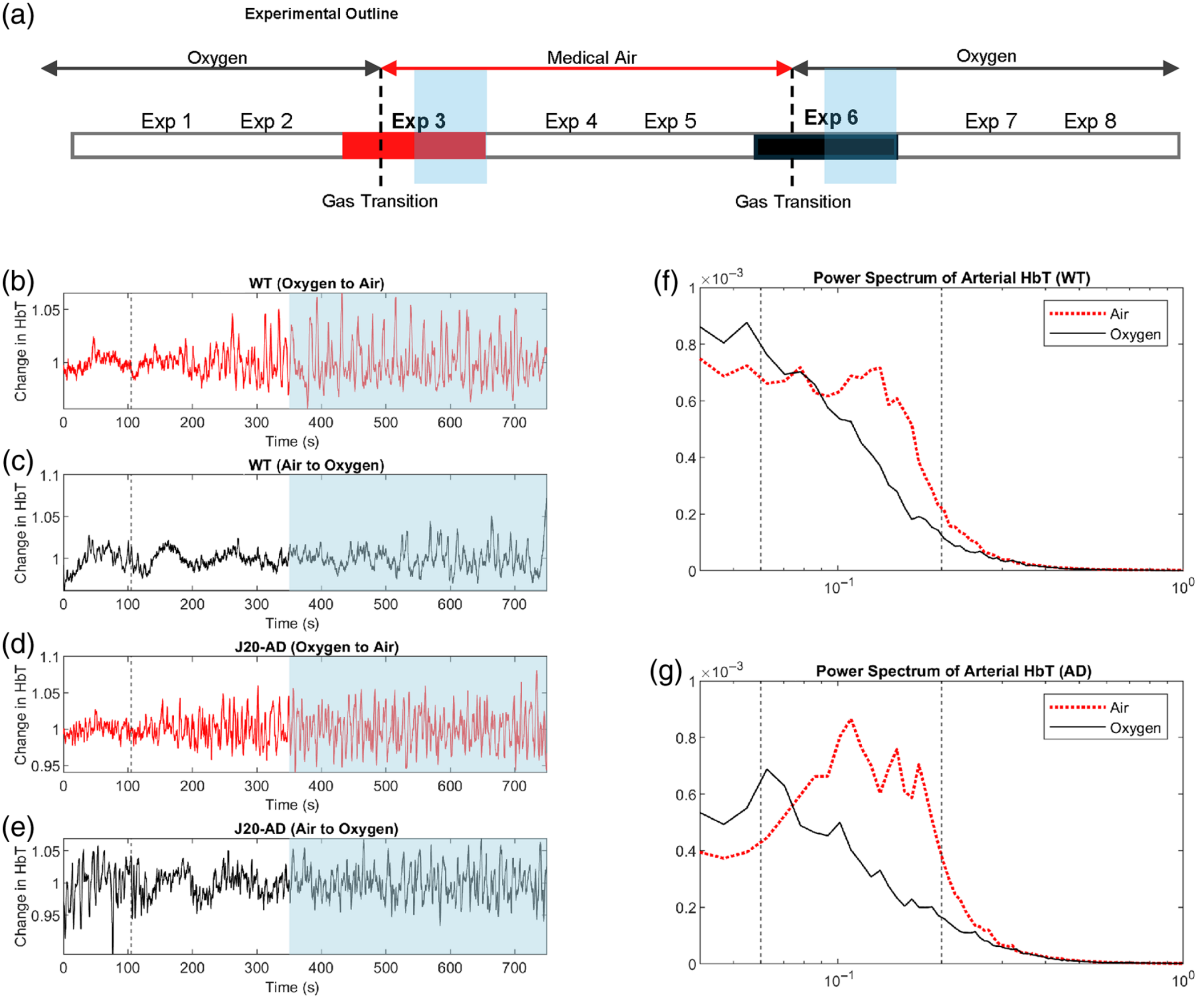
Time series and FFT of arterial HbT. (a) Single session, involving eight experiments. The black dotted lines indicate where inspired gas had been changed. The current study used data from experiments 3 and 6 (marked in bold). The blue highlighted area indicates the portion of the experiment used for the analysis of HbT in each inspired gas condition. Time series of arterial HbT occurring in a WT mouse in (b) an experiment in which the inspired gas was switched from 100% oxygen to medical air, and (c) an experiment in which the inspired gas was switched from medical air to 100% oxygen. Time series of arterial HbT occurring in a J20-AD mouse during (d) an experiment in which the inspired gas was switched from 100% oxygen to medical air, and (e) an experiment in which the inspired gas was switched from medical air to 100% oxygen. The grey dotted line indicates when the inspired gas was changed (105 s), and blue highlighting indicates the portion of data used for the measurement of HbT for each inspired gas condition. Mean FFTs of HbT in the artery for both the oxygen (black) and air-breathing (red) conditions are shown for (f) WT and (g) J20-AD mice; the grey dotted lines indicate the 0.06 to 0.2 Hz range summed and used for analysis.

**Fig. 4:**
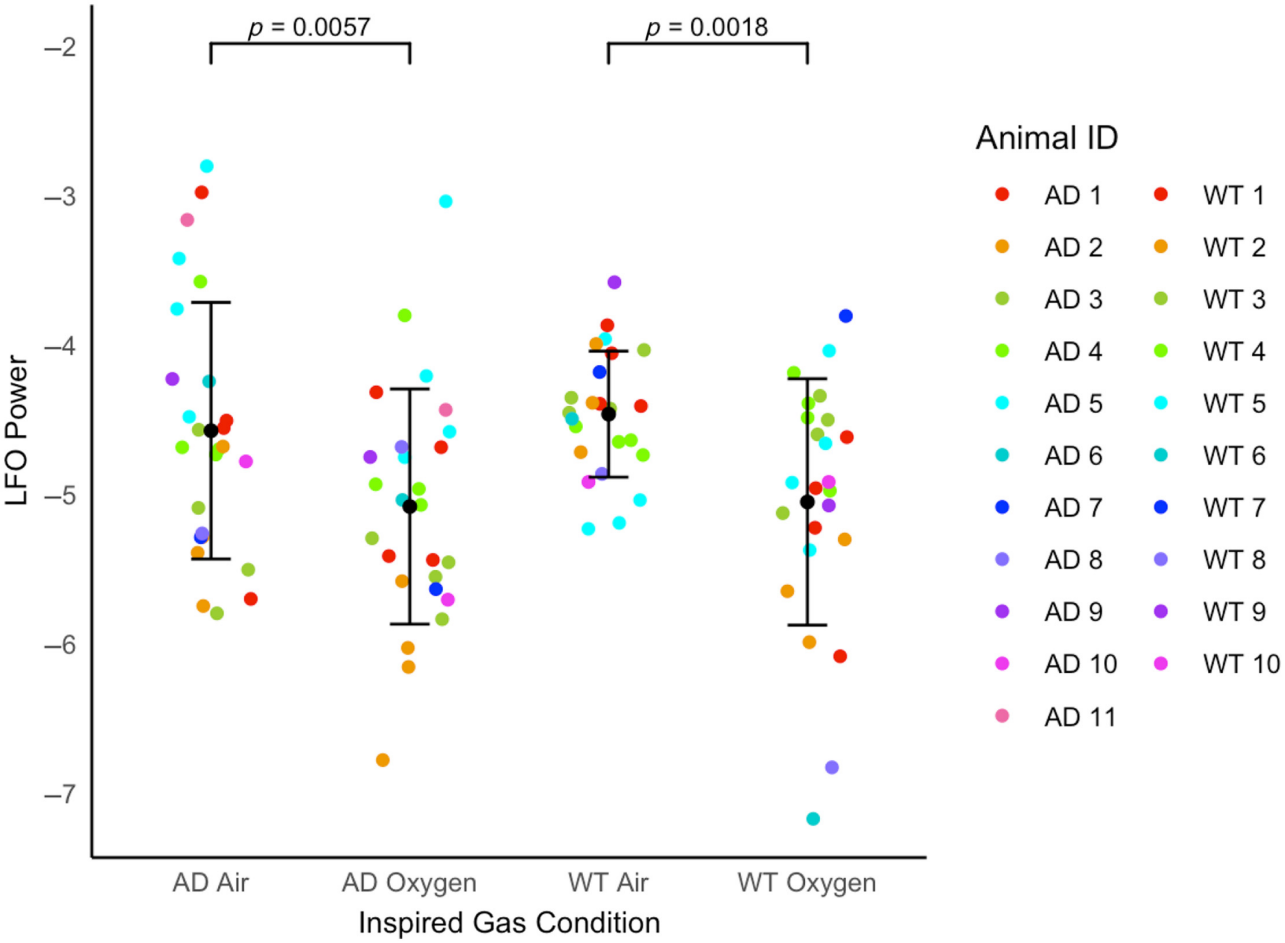
Power of LFOs in the artery of J20-AD and WT mice in oxygen and air-breathing conditions. Log-transformed powers of LFOs occurring in the 0.06 to 0.2 Hz range in the arterial region of J20-AD (n=11) and WT (n=10) mice in each inspired gas condition (oxygen and air). Each mouse contributed no more than four sessions. Each data point represents the summation of power occurring between 0.06 and 0.2 Hz in a session, with mean ± standard deviation (SD) for each group and inspired gas condition.

An FFT was conducted on HbT data from WT and J20-AD mice in the oxygen and air-breathing conditions (Fig. 3), after which the power of the signal occurring in the range of 0.06 to 0.2 Hz was summed and used as a measure of LFOs occurring in the artery. Data were sorted into four groups for analysis using linear mixed modeling: J20-AD oxygen, J20-AD air, WT oxygen, and WT air. The LMM analysis revealed a significant difference in LFO power between these groups [F(3,30.20)=9.05, p<0.001], specifically, there was a significant difference in LFO power between J20-AD mice in the oxygen condition compared with the air condition, as well as a significant difference in LFO power between WT mice in the oxygen condition compared with the air condition, as determined by pairwise comparison (see Table S1 in the Supplementary Material). In both J20-AD and WT mice, LFO power was found to be significantly increased when mice were breathing air compared with breathing oxygen (Fig. 4). However, no significant differences were observed in LFO power between J20-AD and WT mice.

#### 3.1.1 Arterial Low-Frequency Oscillations Are Impaired in J20-AD Mice Compared with WT Controls in the Acute Imaging Session (with Electrode Implanted)

**Fig. 5:**
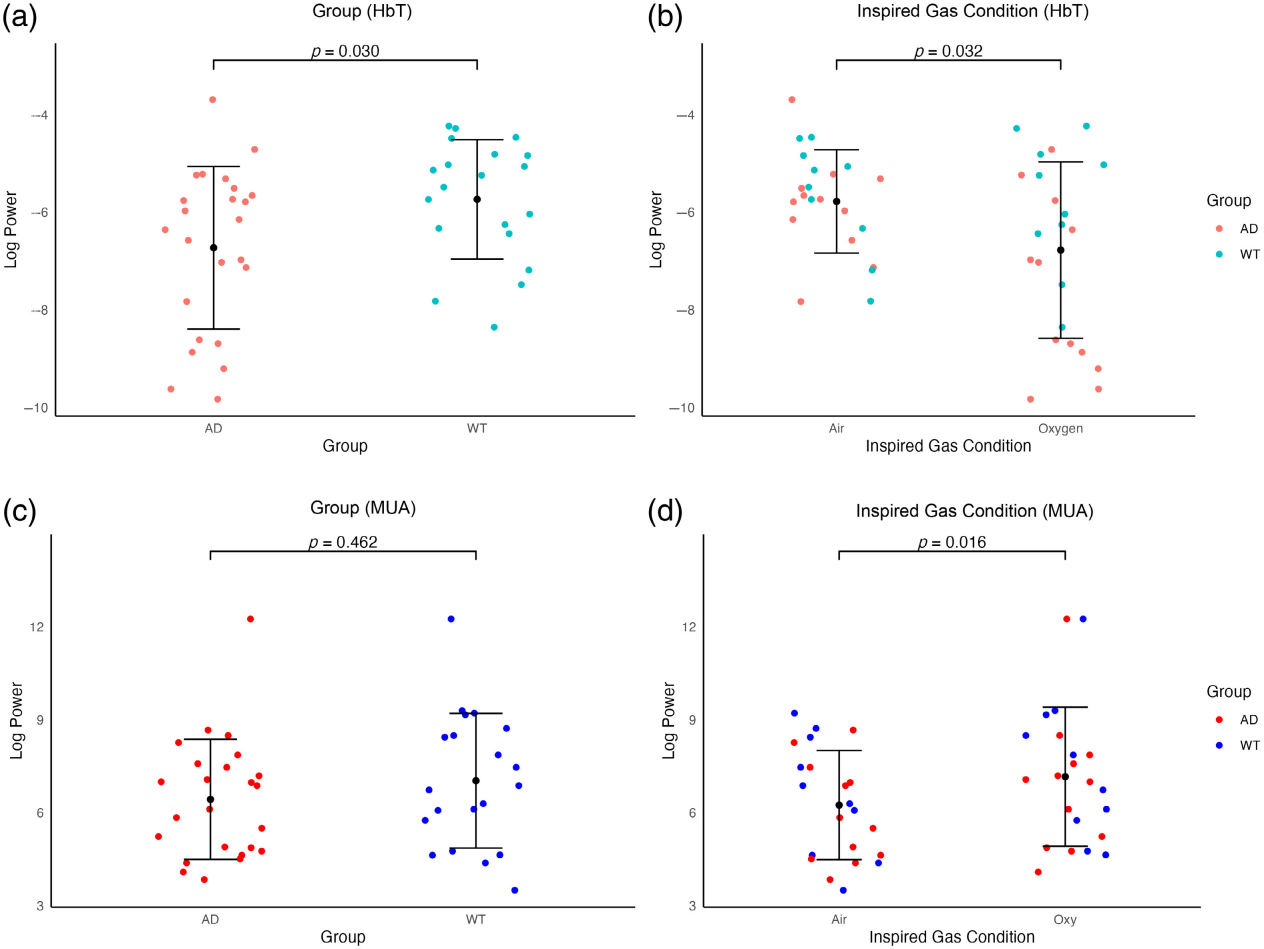
Power of LFOs in the artery of J20-AD and WT mice (with electrode implant) in oxygen and air-breathing conditions. Log-transformed powers of LFOs in HbT occurring in the 0.06 to 0.2 Hz range in the arterial region of (a) each group [J20-AD (n=12) and WT (n=10) mice], and (b) in each inspired gas condition (oxygen and air). Animals contributed one session to each of the inspired gas conditions. Each data point represents the summation of power occurring between 0.06 and 0.2 Hz in a session, with mean ± standard deviation (SD) for each group and inspired gas condition. Log-transformed powers of low-frequency MUA oscillations occurring in the 0.06 to 0.2 Hz range in the somatosensory region of (a) J20-AD and WT mice (with electrode implant), and (b) power of MUA in the somatosensory region of J20-AD and WT mice (with electrode implant) in each inspired gas condition (oxygen and air). Data are plotted as individual values with mean ± standard deviation (SD).

As we wanted to investigate whether HbT LFOs are driven by neural activity, data from mice during the acute session in which an electrode was in place were analyzed. First, these data were analyzed to determine the effect of group (J20-AD or WT C57Bl/6 control) and inspired gas (oxygen or air) on the power of HbT LFOs in the artery ROI. A mixed ANOVA revealed that in the acute dataset, group differences were more pronounced. Specifically, we observed a significant main effect of group [F(1,20)=5.43, p=0.030)], such that J20-AD mice exhibited overall lower power of LFOs than WT controls [Fig. 5(a)]. Similar to the chronic dataset, a significant effect of inspired gas was observed [F(1,20)=5.30, p=0.0320], in that LFOs were found to increase as inspired oxygen was reduced [Fig. 5(b) and Table S2 in the Supplementary Material].

#### 3.1.2 MUA

In the ideal case, changes in inspired gas would affect only neural-independent vasomotion, without altering neural activity. Under such conditions, LFOs in MUA would be expected to remain consistent across breathing conditions. However, a mixed ANOVA on low-frequency MUA revealed significantly higher MUA power during the oxygen condition compared with the air condition (F(1,20)=6.864, p=0.891), with no significant differences observed across groups (Table S2 in the Supplementary Material, Fig. 5). These findings suggest that the LFOs observed in Fig. 5 may result from spontaneous NVC, or from a combination of NVC and neural-independent vasomotion.

#### 3.1.3 Neurovascular coupling

Variations in neural activity across different breathing conditions complicated the interpretation of LFOs in HbT. To address this, we investigated how inspired gas and group influence the relationship between neural activity and hemodynamic responses.

**Fig. 6:**
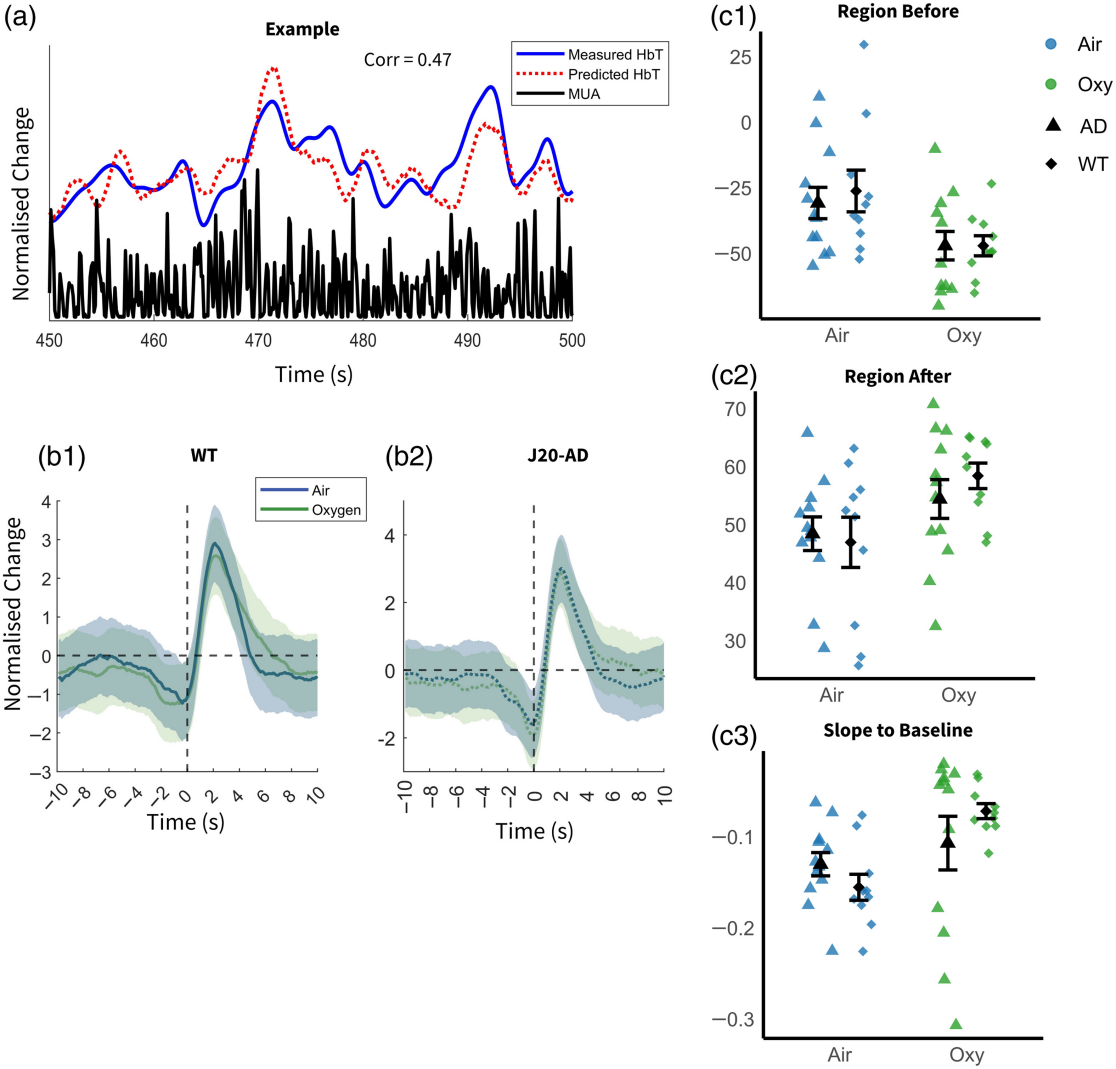
Kernel of MUA and HbT in the artery of J20-AD and WT mice (acute dataset) in oxygen and air-breathing conditions. (a) The HbT and MUA data from a representative subject, over 50 s. (b) The average kernel for WT (b1) and J20-AD (b2) groups. The green line represents the oxygen-breathing condition, whereas the blue line represents the air-breathing condition. The shaded regions denote the standard deviation. (c) The region before (c1), region after (c2), and slope to baseline of all subjects, plotted as individual values with mean ± standard deviation (SD). The green and blue represent air and oxygen-breathing conditions, whereas the square and diamond represent AD and WT mice groups.

To quantify this relationship, we computed a kernel to model the linear relationship between the HbT signal and MUA [i.e., NVC; Fig. 6(b)]. Predicted HbT was obtained by convolving the kernel with MUA data and then correlated with measured HbT [Fig. 6(a)]. Five features of the kernel shape were defined (see Fig. 2), and a mixed ANOVA was applied to analyze these data. To correct for multiple comparisons, a Bonferroni-corrected α-level of 0.01 was applied.

The kernel shape represents the linear relationship between HbT and MUA, that is, the NVC. If the inspired gas or disease group alters NVC, significant differences in kernel shape parameters would be expected. The air breathing condition decrease slope to baseline (F(1,20)=9.279, p=0.006) and region before (F(1,20)=12.278, p=0.002) and increase region after (F(1,20)=9.428, p=0.006) were significantly different across breathing conditions (Table S3 in the Supplementary Material). The kernel prediction reflects the extent to which changes in HbT can be explained by MUA. If the LFOs in HbT observed under air are primarily driven by neural-independent vasomotion—or a mixture of spontaneous NVC and vasomotion—then kernel prediction would be expected to be lower in the air group. Interestingly, a significant interaction was found in the correlation between predicted HbT and measured HbT (F(1,20)=6.643, p=0.018), although the main effects of group and inspired gas were not significant (Table S2 in the Supplementary Material). A follow-up simple effect test revealed that the group mean of kernel prediction for oxygen in the WT condition was significantly higher compared with the AD condition (Table S4 in the Supplementary Material; Oxy-WT, M=0.485, SD=0.159; Oxy-AD, M=0.304, SD=0.231). Importantly, no significant differences in kernel prediction were found across breathing conditions in either the WT or AD group, suggesting that LFO HbT changes across breathing conditions are likely neurally driven.

**Fig. 7:**
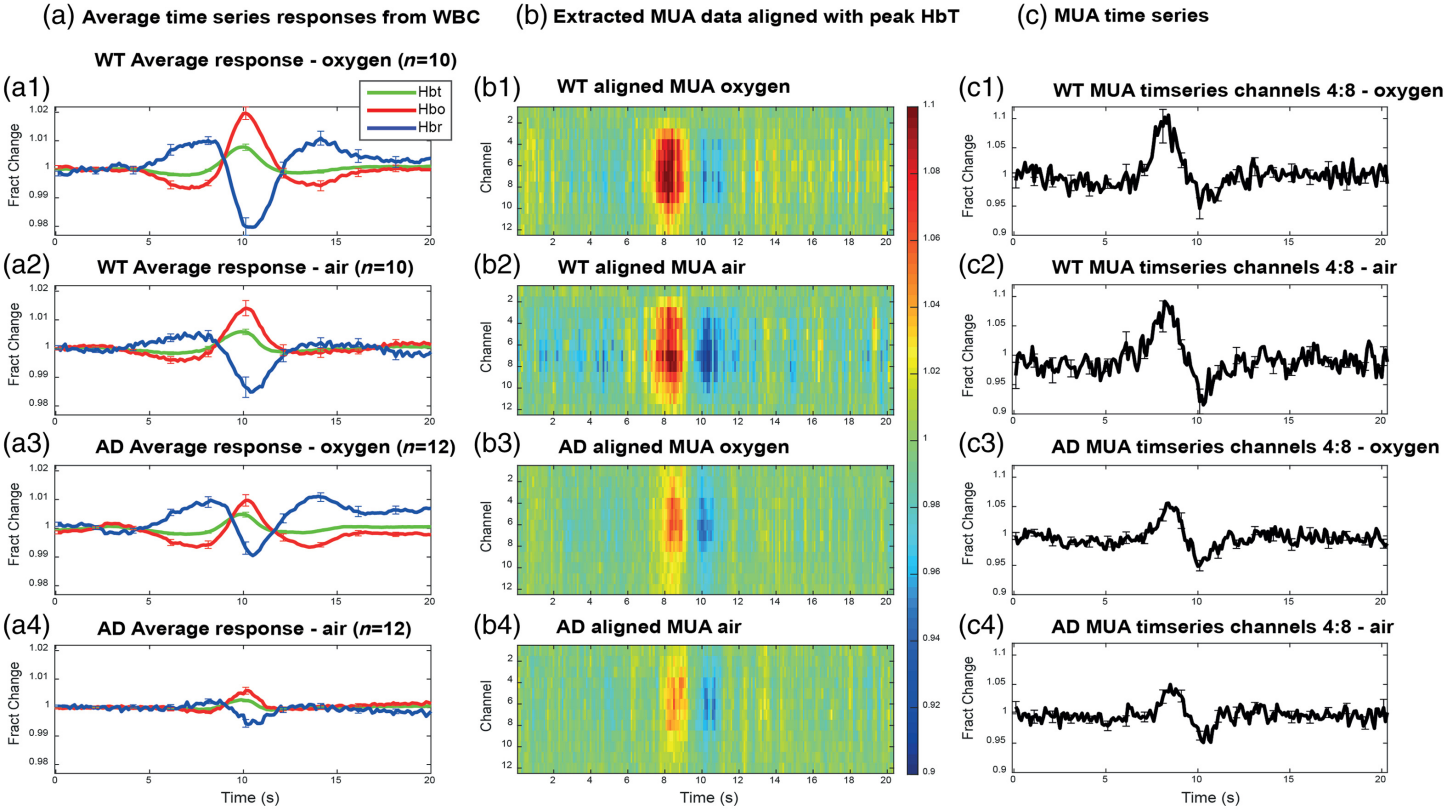
Alignment of spontaneous hemodynamic peak responses and MUA activity across all groups. Column (a) depicts average time series responses from the WBC in (a1) WT animals under oxygen (n=10), (a2) WT animals under air (n=10), (a3) AD animals under oxygen (n=12), and (a4) AD animals under air (n=12). Column (b) depicts extracted MUA data aligned from the peak Hbt response at 10s for (b1) WT animals under oxygen, (b2) WT animals under air, (b3) AD animals under oxygen, and (b4) AD animals under air. The largest increase in MUA activity can be seen at 8 s. The color bar represents fractional change. Column (c) depicts MUA time series taken from channels 4 to 8 in (c1) WT animals under oxygen, (c2) WT animals under air, (c3) AD animals under oxygen, and (c4) AD animals under air. HbT, total hemoglobin; HbO, oxyhemoglobin; HbR, deoxyhemoglobin. Error bars = standard error of the mean.

To further confirm that LFOs in HbT are driven by neural activity, we conducted an additional analysis. We manually identified HbT peaks in WT and AD animals under air and oxygen conditions, extracting corresponding hemodynamic time series for HbT, HbO, and HbR (averaged across animals; Fig. 7). MUA aligned to these hemodynamic peaks was subsequently extracted for neural correlational analysis. If vasomotion was independent of MUA, the expectation would be that the MUA time series would be flat. As can be seen across all groups, the MUA data peak ∼2  s prior to the peak in HbT. Spatially, the MUA data also arise from the middle cortical layers from around the depth of the whisker barrels in layer IV. These data support the results from the kernel analysis, suggesting that, in this study, vascular oscillations were driven by coherent spontaneous neural responses and did not arise independently.

#### 3.1.4 Brain state

To examine whether brain states differ across groups and air-breathing conditions, we employed mixed ANOVA to assess the effects of breathing conditions and group differences on local field potential (LFP) band power across five frequency ranges: delta (0.5 to 4 Hz), theta (4 to 8 Hz), alpha (8 to 12 Hz), beta (12 to 30 Hz), and gamma (30 to 100 Hz). LFP band power was log-transformed to meet normality assumptions. The Bonferroni correction was applied, and the significance level was reduced to 0.01 to account for multiple comparisons.

Significant differences were observed across breathing conditions in the delta, alpha, and gamma bands (Table S5 in the Supplementary Material). Delta and gamma band oscillations are associated with anesthetic state and low-frequency hemodynamic fluctuations, respectively.^50^ In addition, alpha and beta band LFPs differed significantly between groups, consistent with previous findings.^51^

**Fig. 8:**
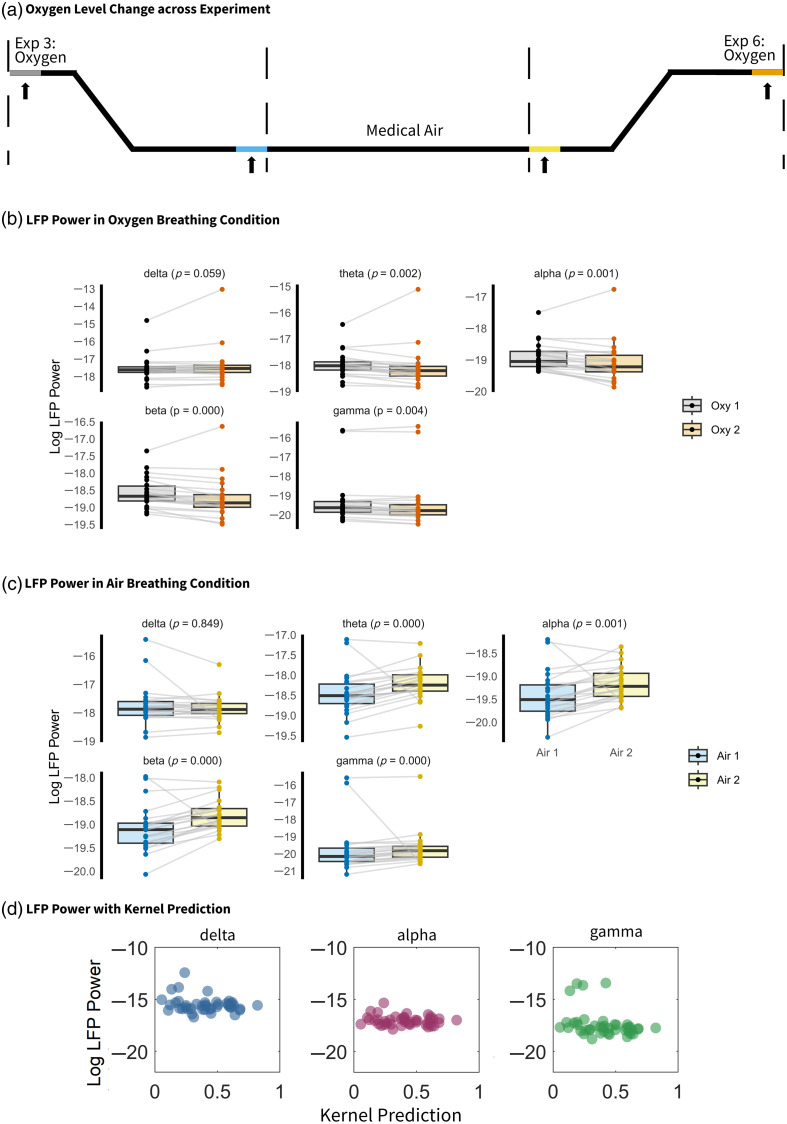
Anesthesia depth remains constant throughout experiments, and kernel prediction is independent of anesthesia depth. (a) Illustration of the experimental setup following electrode implantation. In experiment 3, the breathing condition was switched from oxygen to medical air; in experiment 6, it was switched from medical air to oxygen. The final 450 s of experiments 3 and 6 were used, corresponding to the air and oxygen conditions, respectively. Two 100-s segments from each condition—oxygen (grey and orange) and air (blue and yellow)—were selected for further analysis. The black line represents the oxygen level. (b), (c) LFP power across frequency bands in the two oxygen and air conditions. (d) Log-transformed delta, alpha, and gamma power plotted against kernel prediction.

All experiments were conducted in the same order [see Sec. 2 and Fig. 3(a)]. Therefore, it is crucial to determine whether changes in LFP band power reflect the effects of time or breathing condition. To further examine this, a follow-up ANOVA was performed on LFP power recorded at the beginning of experiment 3 and the end of experiment 6—the two experiments used for analysis in this study (Fig. 8). The results revealed no significant differences in delta-band power between the beginning of Exp. 3 and the end of Exp. 6 [Fig. 8(a), grey and orange; Fig. 8(b)], indicating that anesthetic depth remained stable throughout the recording sessions included in the current analysis. Similarly, delta power in the two air-breathing conditions [Fig. 8(a), blue and yellow; Fig. 8(b)] remained unchanged. This suggests that although breathing conditions may influence delta-band power and, by extension, anesthetic depth, there was no time-dependent effect on anesthetic depth. Furthermore, Fig. 8(d) shows that LFP power in the three frequency bands significantly modulated by gas condition was not correlated with kernel prediction.

## 4 Discussion

The current study found no differences in cortical arterial LFOs between J20-AD and WT mice. These results suggest that measurements of arterial LFOs may not be a suitable metric to distinguish J20-AD males from healthy male controls as differences between diseased and healthy mice were not detected. Furthermore, in line with our hypothesis that a mild respiratory challenge would drive vasomotion oscillations, we found a significant increase in arterial LFO power when inspired oxygen was reduced. It was determined that these LFOs were not entirely independent of neural activity, suggesting that our LFO measurement potentially reflects spontaneous low-frequency NVC rather than vascular-only derived activity.

There were no significant differences in arterial LFOs in HbT in the 0.06 to 0.2 Hz range observed between J20-AD and WT mice in the chronic condition. This aligns with previous findings from our laboratory and others, which found no differences in the neurovascular function of AD mice.^14^^–^^16^ However, when an electrode was inserted into the cortex, group differences were more pronounced. It was revealed that in acute sessions, J20-AD mice exhibited significantly lower powers of arterial LFOs than WT mice when an electrode implant was present in the brain. This aligns with previous literature reporting impaired neurovascular responses after electrode implantation.^15^^,^^16^ This is a result of a CSD, which is a wave of depolarization of neurons and thus silencing of synaptic activity, which may result from electrical or mechanical perturbation of the brain^52^ and is followed by a spontaneous return to normal function after time. The effects of CSD have been reported to be more severe in J20-AD mice, resulting in a larger reduction in HbT and slower hemodynamic recovery than that seen in WT controls.^15^ Thus, group differences in the acute experimental setting of the current study, in which mice underwent intracranial surgery (for electrode insertion) immediately prior to measurements taken of neurovascular function, may have become apparent due to differential effects of CSD on AD and WT mice, in that the J20-AD mice likely exhibited a reduction in HbT that was more prolonged and sustained than that of WT controls. For a more comprehensive report on the differential effects of CSD on J20-AD and WT mice, please see Shabir et al.^15^

The electrode present for acute sessions allowed for the simultaneous measurement of neural activity alongside measurements of changes in cerebral hemodynamics, and this was investigated to see whether the LFOs were driven by neural activity. Significantly higher LFOs in HbT and lower LFOs in MUA were observed under air-breathing conditions. This finding suggested that the manipulation of oxygen concentration influenced neural activity in addition to LFOs in HbT. In the air-breathing experiment (Exp. 6), which always followed the oxygen-breathing experiment (Exp. 3), the observed increase in HbT LFOs may reflect mice recovering from CSD or the presence of neural-independent vasomotion. Furthermore, the MUA power differences suggest the LFOs in HbT across inspired gas conditions could be a result of spontaneous NVC and/or neural independent vascular movement; therefore, further analysis was undertaken to explore the relationship between HbT and neural activity to assess whether any of the hemodynamic LFO increase in air is due to neural-independent vasomotion. Analysis revealed that there were no differences in kernel shape between the groups, consistent with previous studies, which reported that average HbT response to whisker stimulation (i.e., NVC) does not change significantly in J20-AD mice.^15^^,^^16^ As kernel shape reflects the linear relationship between neural activity and the subsequent hemodynamic response, manipulations that evoke changes solely in neural-independent vasomotion should not affect kernel shape. In addition, neural-independent vasomotion is unpredictable based on neural activity; thus, kernel prediction accuracy should decrease with increased neural-independent vasomotion. In the current study, significant differences in kernel shape were found across inspired gas conditions. Analysis revealed a significant interaction between group and inspired gas on kernel prediction despite no significant main effect of group or inspired gas being found. If the breathing condition induces neural-independent vasomotion in both AD and WT mice, we should observe lower kernel prediction in the air compared with the oxygen condition. However, a simple effect test indicated that the mean predictive power in the oxygen-breathing condition is significantly higher in the WT group compared with the J20-AD group, and no significant differences in the kernel prediction across breathing conditions were found. Our additional peak-triggered analysis demonstrated that hemodynamic LFOs consistently followed neural activity (i.e., NVC), as indicated by MUA peaks occurring ∼2  s prior to HbT peaks across all groups and conditions. Collectively, these results suggest that the LFO HbT change induced by oxygen-level change is at least partially neurally induced. Although our findings suggest a linear relationship between LFOs in HbT and MUA, we acknowledge that this approach provides only an indirect assessment of NVC. The NVC responses of the same animals to whisker stimulation have been reported in our previous work.^15^^,^^16^

Interpretations of relationships between neural activity and hemodynamic response data between the WT and J20-AD groups must be approached with caution as electrode implantation induces differential CSD effects in WT compared with J20-AD mice, in that the effects of the CSD appear to be more profound in the J20-AD model than in WT mice.^15^ Future research should prioritize less invasive techniques, such as the use of single-fluorophore genetically encoded calcium indicators (GECIs), such as GCaMP. This allows for *in vivo* imaging of brain activity without the need for an electrode; thus, next steps may be to investigate LFOs in HbT using this technique. This would allow for a better assessment of differences in neural activity and kernel prediction between WT and J20-AD groups.

Classically, vasomotion has been defined as an ∼0.1  Hz oscillation in vessel diameter that occurs independently of neuronal activity.^18^^,^^33^ However, studies in mice have reported instances of entrainment or coupling between vasomotion and neuronal signals,^35^^,^^50^ suggesting a more intricate relationship. In the current study, changes in our LFO signal were seemingly induced by neural activity, contributing to the understanding of the connection between vasomotion and neuronal activity in the mouse. The current study found no differences in LFO power between WT and J20-AD mice, the results of which contrast with studies reporting changes in vasomotion in several disease states, such as hypertension^22^^,^^23^ and AD.^29^^,^^30^ A better understanding of vasomotion in AD is of significant importance as it has been postulated that this process may aid in the clearance of Aβ from the brain,^31^^,^^32^ and thus, could be a key therapeutic target for the prevention or treatment of AD. It has also been suggested that the oscillatory blood flow produced by vasomotion can help achieve better tissue oxygenation,^25^ which aligns with studies reporting higher amplitudes of vasomotion in conditions associated with hypoperfusion.^20^^,^^21^

We had hypothesized that a mild respiratory challenge (inspired gas transition from 100% $O_{2}$ to 21% $O_{2}$) would drive vasomotion oscillations; however, this does not seem to be the case as significant changes in low-frequency MUA and kernel shape were detected across the different inspired gas conditions. Vasomotion oscillations might have been more prominent using methods such as changing arterial pressure^33^ or administering L-NAME,^53^ rather than changing inspired gases, although the aforementioned methods were implemented successfully in rat models, and thus, it may be possible that cerebral vasomotion is more difficult to induce or measure successfully in the mouse. Further research is needed to characterize vasomotion both in the mouse and in the AD state.

Methods such as blood-oxygen level dependent (BOLD) fMRI have been used to study brain activity in AD in both humans^54^^,^^55^ and in animal models^56^^,^^57^ of the disease. It is known that factors such as motion, respiratory and cardiac cycles, and vasomotion can confound BOLD signals,^58^ highlighting the importance of investigating the role of nonneuronal factors such as vascular fluctuations, to better understand how these signals may impact the interpretation of resting-state fMRI data. Our study found that LFOs in arterial HbT were coupled to neuronal activity. This may contribute to the understanding of the resting-state BOLD signal as used in functional connectivity mapping, strengthening the interpretation of the relevant low-frequency vascular oscillations as reflective of underlying neuronal activity. It has been suggested that a dynamic relationship between vasomotion and neuronal activity may exist, in that vasomotion has been reported to be influenced by or tied to underlying neural activity in awake mice;^35^^,^^59^ thus, an avenue for future research may be to explore this relationship further. Ma et al. investigated the relationship between resting-state hemodynamics and underlying neural activity using Thy1-GCaMP mice and wide-field optical mapping and found that Thy1-GCaMP signals were coupled to multiunit activity,^50^ providing further evidence that resting-state hemodynamics may reflect underlying patterns of neural activity.

There are some limitations of the current study to address, one being that the measurements of neurovascular function were taken in animals under anesthesia. It is known that cerebral hemodynamics, neural activity, and NVC in anesthetized animals is different from that of awake animals,^60^^,^^61^ and thus, the state produced by anesthetics does not reflect normal physiological conditions. However, previous research from our laboratory has indicated that the anesthetic combination used in the current study results in a magnitude and speed of evoked hemodynamic responses to physiological whisker stimulation that resemble those found in the awake state.^42^ However, the impact that our anesthetic regime may or may not have on LFOs specifically has not yet been determined. Furthermore, the current experiments did not include the collection of additional physiological monitoring data (e.g., breathing rate, heart rate, and movement) during anesthesia, making it challenging to accurately quantify the anesthetic state. However, we have shown previously that baseline hemodynamic values remain stable throughout the duration of the experiments, apart from time points where an intervention took place to change the baseline state (i.e., gas challenges or hypercapnia), (see figure 5 of Sharp et al.^16^).

To further investigate the anesthetic state of the animals in the present study, LFP power was analyzed. Among the frequency bands, delta-band activity (0.5 to 4 Hz) has emerged as the most reliable indicator of anesthetic depth in the experimental setting.^62^ There was a significant difference in delta-band LFP in air compared with oxygen breathing condition, but no significant differences were observed in the delta band LFP power in the beginning and end of the experimental data that were used in the current study (i.e., the beginning of Exp. 3 and end of Exp. 6). Thus, although breathing condition may influence brain state, no evidence of a time-dependent change in brain state was observed. Furthermore, no significant correlation was found between the fraction of LFO HbT explained by neural activity (as assessed by kernel prediction) and changes in LFP bands across different breathing conditions. Therefore, we suggest that the observed LFP variations across breathing conditions do not undermine the conclusions drawn. Nevertheless, future studies employing anesthetized models should incorporate anesthetic state monitoring and adopt a counterbalanced experimental design.

The current study also cannot quantify whether mice experienced different sleep states while under anesthesia. This may be an interesting avenue for future research as Hauglund et al.^63^ demonstrated that norepinephrine-mediated vasomotion occurs during NREM sleep and may also aid in driving glymphatic clearance. Another limitation of the current study is that the measurements taken for neurovascular function in the oxygen and air-breathing conditions were taken at different points of time following electrode insertion (i.e., experiments 3 and 6 of eight total experiments). This means that recovery after electrode insertion will vary slightly, in that measurements taken in the later session would have been after the animals had more time to recover following the CSD, and thus, NVC responses may not be as diminished as those observed in the earlier experiment (i.e., in experiment 3). All animals experienced one CSD event immediately following electrode insertion, the hemodynamic effects of which persisted for some time before recovery^15^ (see Shabir et al.). However, electrode insertion occurred 30 min prior to experiment 1, meaning that experiment 3 took place ∼1  h after insertion, which should be sufficient time to allow for hemodynamic recovery, at least in healthy mice.^15^ In addition, it should be noted that all mice included in the current study were male; thus, our findings do not account for any potential sex differences. As female mice often exhibit a more severe AD phenotype,^64^^,^^65^ an investigation into arterial LFOs in female mice would be an important avenue for future research. Future work may also benefit from the use of an imaging apparatus with greater spatial resolution and depth specificity than that which can be achieved by OIS imaging, such as two-photon microscopy, to potentially provide more precise insights into the relationship between cerebral hemodynamics and neural activity. This could also allow for the quantification of diameter fluctuations in cerebral arterial vessels, which would be valuable in the investigation of cerebral vasomotion *in vivo*.

### 4.1 Conclusion

The current study found no differences in arterial LFOs of J20-AD and healthy WT mice, except for in our acute preparation in which an electrode implant was present, likely due to the effects of a CSD from electrode insertion. Thus, measurements of LFOs in the artery may not be a feasible biomarker to distinguish between diseased and healthy states in male J20-AD mice. We had hypothesized that a mild respiratory challenge would drive vasomotion oscillations, and we observed an increase in LFO power as inspired oxygen was reduced. Upon further analysis, it was revealed that these LFOs were not independent of neuronal activity. Our results suggest that low-frequency vascular oscillations may be driven by spontaneous neural activity, thus adding to the understanding of how vascular fluctuations may contribute to the resting-state BOLD response. The current study also revealed that hemodynamic oscillations occurring within the same frequency range as vasomotion were coupled to neuronal activity, providing evidence that a dynamic relationship might exist between vasomotion and neuronal activity in the mouse. Further work in this area is needed, both to obtain a better understanding and characterization of vasomotion in AD and to find potential vascular biomarkers for the early detection of the disease.

## 5 Supplementary Material

10.1117/1.NPh.12.S1. S14615.s01

## Data Availability

Data presented in this paper are available on the Dryad Digital Repository: https://doi.org/10.5061/dryad.q83bk3jt4.
