# Supplementary material for: Hemodynamic and neuronal contributions to low-frequency vascular oscillations in a preclinical model of Alzheimer’s disease
Source: Neurophotonics. 2025 Jul 22;12(Suppl 1):S14615. doi: 10.1117/1.NPh.12.S1.S14615 (PMC12285523; doi:10.1117/1.NPh.12.S1.S14615)
Supplement: Supplementary file 1 [file NPh_012_S14615_SD001.pdf]

Supplementary Material

Table S1 Linear Mixed Model - Pairwise Comparisons

	b	SE	t	p
AD Air vs. AD O2	0.5076	0.146	3.485	0.0057*
AD Air vs. WT Air	-0.0367	0.279	-0.131	0.9992
AD Air vs. WT O2	0.5506	0.279	1.974	0.2255
AD O2 vs. WT Air	-0.5442	0.279	-1.951	0.2342
AD O2 vs. WT O2	0.0431	0.279	0.154	0.9986
WT Air vs. WT O2	0.5873	0.152	3.874	0.0018*

Note. significant terms are denoted with an asterisk. P-values are Tukey-adjusted for multiple comparisons.

Table S2 ANOVA

	**Inspired Gas (Oxygen vs Air)**			**Group (J20-AD vs WT)**			**Inspired Gas*Group**		
	F(1,20)	p	η2	F(1,20)	p	η2	F(1,20)	p	η2
HbT (Acute)	5.30	.032*	.209	5.43	.030*	.213	3.62	.072	.153
MUA	6.864	.016*	.052	.562	.462	.023	.007	.934	.056
Kernel Prediction	.019	.891	.000	.775	.389	.026	6.643	.018*	.091

Note. ‘HbT’ in the table refers to power of LFOs in HbT within the 0.06-0.2 Hz range. Significant terms are denoted with an asterisk.

Table S3 ANOVA with Multiple Comparison on Kernel Shape Parameters

	**Inspired Gas (Oxygen vs Air)**			**Group (J20-AD vs WT)**			**Inspired Gas*Group**		
	F(1,20)	p	η^2	F(1,20)	p	η^2	F(1,20)	p	η^2
Slope to Baseline	9.279	.006*	.162	.056	.815	.002	2.969	.100	.058
Region Before	12.278	.002*	.194	.119	.733	.004	0.189	.668	.004
Region After	9.428	.006*	.150	.117	.736	.004	.931	.346	.017
Peak	1.437	.245	.025	0.145	.707	.005	0.219	.645	.004
	Q(1,20)	p	η2	Q(1,20)	p	η2	Q(1,20)	p	η2
Slope to Peak	.029	.869	.003	2.101	.173	.151	.051	.825	.005

Note. significant terms are denoted with an asterisk.

Table S4 Simple Effects Test on Kernel Prediction

	Contrast	estimate	p	t- ratio
J20-AD	Inspired Gas	.126	.125	1.566
WT	Inspired Gas	-.113	.207	-1.284
Air	Group	.058	.499	.682
Oxygen	Group	-.182	.037*	-2.152

Note. significant terms are denoted with an asterisk.

Table S5 ANOVA with Multiple Comparison on LFP Band Powers

	**Inspired Gas (Oxygen vs Air)**			**Group (J20-AD vs WT)**			**Inspired Gas*Group**		
	F(1,20)	p	η^2	F(1,20)	p	η^2	F(1,20)	p	η^2
Log-delta	9.851	.005*	.033	2.950	.101	.121	<.001	.994	<.001
Log-theta	6.818	.021	.026	6.283	.017	.225	.092	.764	<.001
Log-alpha	14.188	.001*	.047	8.139	.010*	.275	.161	.692	<.001
log-beta	21.135	<.001*	.075	11.771	.003*	.352	.029	.867	<.001
log-gamma	35.697	<.001*	.010	3.548	.074	.150	.609	.444	<.001

Note. significant terms are denoted with an asterisk.
